# Renal safety and survival among acutely ill hospitalized patients treated by blockers of the Renin-Angiotensin axis or loop diuretics: a single-center retrospective analysis

**DOI:** 10.1080/0886022X.2023.2282707

**Published:** 2023-11-17

**Authors:** May Assaly, Yuri Gorelik, Samuel N. Heyman, Zaid Abassi, Mogher Khamaisi

**Affiliations:** a Department of Medicine D, Rambam Health Care Campus, Haifa, Israel; b Department of Medicine, Hadassah Hebrew University hospital, Jerusalem, Israel; c Department of Physiology, Bruce Rappaport School of Medicine, Technion, Haifa, Israel

**Keywords:** Acute kidney failure, renal functional reserve, angiotensin-receptor blockers, converting-enzyme inhibitors, furosemide, chronic kidney disease, hypotension

## Abstract

**Background:**

Concern exists regarding the renal safety of blocking the renin-angiotensin system (RAS) during acute illness, especially in the presence of volume depletion and hemodynamic instability.

**Methods:**

We explored the impact of loop diuretics and RAS blockers on the likelihood of developing acute kidney injury (AKI) or acute kidney functional recovery (AKR) among inpatients. Adjusted odds ratio for AKI, AKR and mortality was calculated, using logistic regression models, with subgroup analysis for patients with estimated glomerular filtration rate (eGFR) <30 ml/min/1.73 m2, corrected for blood pressure measurements.

**Results:**

53,289 patients were included. RAS blockade was associated with reduced adjusted odds ratio for both AKI (0.76, CI 0.70–0.83) AKR (0.55, 0.52–0.58), and mortality within 30 days (0.44, 0.41–0.48), whereas loop diuretics were associated with increased risk of AKI (3.75, 3.42–4.12) and mortality (1.71, 1.58–1.85) and reduced AKR (0.71, 0.66–0.75). Comparable impact of RAS blockers and loop diuretics on renal outcomes and death was found among 6,069 patients with eGFR < 30 ml/min/1.73m^2^. RAS inhibition and diuretics tended to increase the adjusted odds ratios for AKI and to reduce the likelihood of AKR in hypotensive patients.

**Conclusions:**

Reduced blood pressure, RAS blockers and diuretics affect the odds of developing AKI or AKR among inpatients, suggesting possible disruption in renal functional reserve (RFR). As long as blood pressure is maintained, RAS inhibition seems to be safe and renoprotective in this population, irrespective of kidney function upon admission, and is associated with reduced mortality.

## Introduction

The renin-angiotensin-aldosterone system (RAS) plays a central role in human diseases, promoting vasoconstriction and fibrosis. Its inhibition by angiotensin-converting enzyme inhibitors (ACEi) and angiotensin-AT1-receptor blockers (ARBs) or through the interference with aldosterone action revolutionized the management of cardiovascular and renal disorders [1,2]. ACEi and ARBs attenuate the progression of chronic kidney disease (CKD) [3,4]. Renoprotection is believed to be mediated in part by vasodilation of glomerular efferent arterioles, with reduction of trans-glomerular pressure, diminished proteinuria, and, consequently, attenuating the progress to glomerulosclerosis and tubulo-interstitial fibrosis [5]. Reduced GFR and downstream tubular transport improves medullary hypoxia and likely also attenuates mal-adaptive hypoxia-driven and RAS-mediated interstitial fibrosis [6].

The impact of RAS blockade has also been extensively studied in patients following hospitalization for an acute illness with- or without AKI [7–11]. Despite some conflicting outcomes, overall these studies suggest reduced mortality and a long-term renal protection. Yet, the safety of RAS inhibition remains debated during hospitalization and under acute settings, especially among patients with advanced CKD [12]. For instance, concerns have been raised regarding the continuation of RAS inhibition throughout elective surgery, particularly cardiovascular procedures, with potential hemodynamic instability and hypotension. Indeed, pre-operative discontinuation of ACEi was found to attenuate perioperative decline in blood pressure and the need for pressors, arguing for AKI prevention by pre-operative withholding of RAS blockers, particularly in patients undergoing cardiac surgery [13–16]. The COVID-19 epidemic invoked an additional unique argument regarding the safety of RAS inhibitors during an acute illness. Cell-membrane-bound angiotensin converting enzyme (ACE)2 serves as the viral homing target, and as its expression increases with RAS inhibitors, concern was raised regarding enhanced viral cell-attachment and invasion [17]. On the other hand, ACE2 depletion in this disorder compromises the ACE-2/Ang 1-7/MasR vasodilating and tissue-protective axis, that counterbalances the ACE/Ang II/AT1R vasoconstrictive, pro-inflammatory and pro-coagulant axis. Consequently, continuing RAS blockers has been recommended in patients with COVID-19 disease [18]. Hypothetically, ARBs might be superior to ACEi, since Ang II serves as the substrate for ACE2, being converted into Ang 1-7. Indeed, a retrospective analysis of 625 patients on RAS blockers with severe COVID-19 disease showed that continuation of ARBs was associated with substantially improved outcome, as compared with patients who were kept on ACEi [19]. In another study, uninterrupted administration of RAS inhibitors in hypertensive patients hospitalized with COVID-19 disease was associated with a better prognosis as long as hypotension or AKI were absent [20].

Surprisingly, while pros and cons regarding RAS blockade have been explored in clinical trials of elective surgery and in patients infected with SARS-CoV-2, the renal safety of RAS inhibition has not been studied in depth in non-surgical patients with an acute illness requiring hospitalization, and concern remains regarding RAS inhibition, especially in the presence of volume depletion and hemodynamic instability. Herein we report a retrospective analysis of changes in serum creatinine (sCr) among inpatients in departments of Medicine, analyzing the independent impact of RAS blockers and loop diuretics during the early hospitalization course, corrected for blood pressure measurements, on the likelihood of developing AKI or acute kidney functional recovery (AKR). This later parameter, mostly reflecting resolving AKI, is common among hospitalized patients [21], and until recently has been totally ignored.

## Methods

We conducted a single-center retrospective analysis, using the MDClone platform system, as previously performed [22–24]. The study was performed in adherence to the Declaration of Helsinki and approved by the institutional review board at Rambam Health Care Campus (IRB RMB-D-0195-21). Included were all adult (>18 y) patients hospitalized in the departments of Medicine at Rambam health care campus, Haifa, Israel during the years 2012–2022, with available sCr upon admission or 72 h before hospitalization and repeated subsequent measurements within 72 h after admission. For patients with recurring hospitalizations, the first hospitalization only with the required creatinine measurements was included. Patients on dialysis on admission were excluded.

Multiple variables in the patients’ data base were evaluated, including demographics, medical diagnoses, hemodynamic parameters, laboratory values and medications administered during the hospital stay. Medications were identified and grouped by their Anatomical Therapeutic Chemical classification. Specifically, we analyzed individually various hypertensive medications and loop diuretics given during the early hospitalization course, and analyzed RAS inhibitors (ACEi, ARBs and angiotensin receptor-neprilysin inhibitor [ARNI]) grouped together. Medical conditions were identified and grouped by their International Classification of Diseases (ICD) codes. For continuous and categorical data, only variables that were available for at least 75% of patients and were positive in at least 5% of the patient population, respectively, were included. Only patients with available data for at least 75% of the variables were included in the final analysis. We impute all missing information with median values for continuous variables and with the common value for dichotomous variables.

Exposure to contrast medium included all patients who received iodinated radiocontrast material up to 2 days following admission. Blood pressure values represent medians of repeated measurements over 72h from admission. Estimated glomerular filtration rate (eGFR) was calculated according to the Chronic Kidney Disease Epidemiology Collaboration (CKD-EPI) equation.

Primary outcome measures were AKI and AKR. AKI was diagnosed when the difference between sCr at the time of admission and the first creatinine measured within 24–72 h of admission fulfilled the Kidney Disease Improving Global Outcomes (KDIGO) definition of AKI [25]. AKR, meaning recovering from AKI present on admission (evidenced by declining sCr along time), was identified and classified as a mirror image of AKI definitions, namely when sCr upon admission fell along time by 0.3 mg/dl or more, or was at least 1.5-fold higher than subsequent creatinine measurements obtained 24–72 h post admission [22,23]. Focusing on the very first period following admission, and in order to avoid the impact of later confounders along the hospitalization course, we did not study changes in kidney function beyond 72 h, nor did we analyze subsequent changes in medications. All-cause mortality within 30 days of admission, retrieved from the National Registry, was defined as a secondary outcome measure.

All statistical analyses were performed using the R version 4.2.0 (R Foundation for Statistical Computing). Baseline variables (on admission) were compared between patients on- or without anti-hypertensive and diuretic agents, using the Mann-Whitney U test for continuous variables and presented as medians with inter quartile ranges, and categorical variables were compared, using the chi-square test and presented as absolute numbers and percentages.

To study the effect of various anti-hypertensive agents on primary and secondary defined outcomes we used a multivariable logistic regression model for each outcome variable. Most commonly used anti-hypertensive agents were incorporated as primary exposure variables, secondary effects included parameters found to be associated with changes in renal function among inpatients, as indicated in our previous studies [22–24]. The covariates that were used in the regression model are listed in Tables 2 and 4. We used cubic splines to plot the predicted probabilities of AKI and AKR as a function of baseline systolic blood pressure as calculated by the regression models, with the Hosmer-Lemeshow test used to assess the model’s goodness of fit with partitioning defined to 10 groups.

**Table 1.** Demographic and clinical characteristics of all included patients, also stratified by the administration of anti-hypertensive medications and loop diuretics during hospitalization.

Variable	All patients (*n* = 53,289)	Anti-HTN/diuretic Tx (*n* = 34,615)	Control untreated (*n* = 18,674)
Age, years (medians with inter quartile ranges)	67.3 [55.2, 80.2]	74 [63.9, 82.6]	55.1 [37.8, 70.3]
Male, n (%)	27,495 (51.5%)	17,518 (51.7%)	9,977 (53.4%)
Diagnoses, n (%)			
CKD	1,865 (3.5%)	1,650 (4.8%)	215 (1.2%)
Diabetes mellitus type II	16,477 (30.9%)	13,927 (40.2%)	2,550 (13.7%)
Hypertension	28,217 (52.9%)	24,990 (72.2%)	3,227 (17.3%)
CHF	6,449 (12.1%)	6,225 (18%)	224 (1.2%)
Atrial fibrillation	7,604 (14.2%)	7,164 (20.7%)	440 (2.4%)
Multiple myeloma	351 (0.6%)	242 (0.7%)	109 (0.6%)
ACS	2,528 (4.8%)	2,404 (7.1%)	124 (0.7%)
Acute infection	12,400 (23.2%)	7,516 (21.9%)	4,884 (26.2%)
Medications and interventions during hospitalization, n (%)			
RAS inhibition, combined	19,573 (36.7%)	19,573 (56.5%)	0
ACE inhibitors	14,004 (26.2%)	14,004 (40.4%)	
ARBs	6,318 (11.8%)	6,318 (18.2%)	
Renin inhibitor	6 (0%)	6 (0%)	
ARNI	75 (0.1%)	75 (0.2%)	
Beta blockers	23,166 (43.4%)	23,166 (66.9%)	0 (0%)
Loop diuretics	12,955 (24%)	12,955 (37%)	0 (0%)
CCB	13,927 (26%)	13,927 (40%)	0 (0%)
Contrast administration	8,711 (16.3%)	5,302 (15%)	3,409 (18.3%)
Packed red blood cells administration	3,088 (5.7%)	1,897 (5.5%)	1,191 (6.4%)
Vital signs (medians with inter quartile ranges)			
Systolic blood pressure, mmHg	127.4 [112, 144]	132 [117, 150]	119 [107, 132]
Diastolic blood pressure, mmHg	69.6 [64, 77]	70 [64, 78]	69 [63, 75]
Mean blood pressure, mmHg	88.7 [80, 99]	90.6 [81, 102]	85.6 [77, 94]
Heart rate, bpm	77.7 [69, 88]	76 [67, 86]	81 [72, 90]
Oxygen saturation, %	96.3 [95, 98]	96 [95, 98]	97 [95, 99]
Temperature, °C	37.2 [37, 37.9]	37.2 [37, 37.8]	37.3 [37, 38.2]
Laboratory results (medians with inter quartile ranges)			
Admission creatinine, mg/dL	0.93 [0.8, 1.3]	1 [0.8, 1.4]	0.8 [0.7, 1.1]
BUN, mg/dL	21.2 [13, 28]	24 [15, 31.6]	16 [10, 20]
Potassium, mmol/L	4 [3.7, 4.3]	4 [3.7, 4.3]	3.9 [3.6, 4.2]
Sodium, mEq/L	137 [135, 139]	137 [135, 139]	137 [135, 139]
Total protein, g/dL	6.6 [6, 7.1]	6.6 [6, 7.1]	6.5 [5.9, 7]
Albumin, g/dL	3.3 [2.9, 3.8]	3.3 [2.9, 3.7]	3.3 [2.8, 3.8]
CRP, mg/dL	9.2 [2.1, 25.5]	8.8 [2, 24.7]	9.7 [2.2, 26.6]
Blood pH	7.4 [7.3, 7.4]	7.4 [7.3, 7.4]	7.4 [7.3, 7.4]
Lactate, mmol/L	2.1 [1.5, 2.9]	2.1 [1.5, 2.9]	2 [1.4, 2.9]
Hemoglobin, mg/dL	11.5 [10.3, 13.5]	11.5 [10.3, 13.4]	11.6 [10.3, 13.6]
White blood cells, k/mcL	10 [6.6, 11.7]	9.9 [6.8, 11.6]	10.2 [6.3, 12]
Platelets, k/mcL	210 [161, 269]	209 [162, 266]	212 [159, 276]
Outcome, n (%)			
AKI grade 1	2784 (5.2)	2302 (6.7)	482 (2.6)
AKI grades >1	223 (0.4)	154 (0.4)	69 (0.4)
AKR grade 1	9698 (18.2)	6265 (18.1)	3433 (18.4)
AKR grades >1	482 (0.9)	264 (0.8)	218 (1.2)
Dialysis within 30 days	175 (0.3)	144 (0.4)	31 (0.2)
Mortality within 30 days	4566 (8.6)	3096 (8.9)	1470 (7.9)

Blood pressure values represent medians of repeated measurements over 72h from admission. All other vital signs and laboratory results are those on admission. HTN: hypertension; CKD: chronic kidney disease; CHF: congestive heart failure; ACS: acute coronary syndrome; RAS: renin-angiotensin system; ACE: angiotensin converting enzyme; ARB: angiotensin receptor blocker; ARNI: angiotensin receptor-neprilysin inhibitor; CCB: calcium channel blockers; BUN: blood urea nitrogen; CRP: C-Reactive protein.

**Table 2.** Adjusted odds ratios of variables and interaction terms for the logistic regression models of acute kidney injury (AKI), acute kidney recovery (AKR) and mortality in 53,289 patients.

Adjusted odds ratio (95% confidence interval)			
	AKI	AKR	Mortality
Beta blockers	1.11 (1.02–1.22)	0.88 (0.85–0.94)	0.73 (0.68–0.79)
RAS inhibitors	0.76 (0.7–0.83)	0.55 (0.52–0.58)	0.44 (0.41–0.48)
Loop diuretics	3.75 (3.42–4.12)	0.71 (0.66–0.75)	1.71 (1.58–1.85)
Contrast administration	1.04 (0.92–1.17)	0.67 (0.62–0.72)	1.25 (1.14–1.37)
Male gender	0.99 (0.91–1.07)	1.23 (1.18–1.29)	1.11 (1.04–1.18)
Age (per increase of 10 years)	1.25 (1.21–1.28)	1.22 (1.2–1.24)	1.73 (1.69–1.77)
Diabetes mellitus	1.3 (1.2–1.41)	1.64 (1.56–1.72)	1.17 (1.09–1.26)
Atrial fibrillation	0.9 (0.81–1.00)	0.92 (0.86–0.99)	0.91 (0.83–0.99)
CHF	1.09 (0.98–1.21)	1.13 (1.04–1.22)	0.92 (0.83–1.02)
CKD	1.80 (1.55–2.08)	1.47 (1.32–1.64)	1.10 (0.94–1.27)
Minimum SBP (per increase of 10 mmHg)	0.96 (0.95–0.98)	0.96 (0.95–0.97)	0.88 (0.87–0.89)
Acute infection	1.42 (1.30–1.55)	1.66 (1.58–1.75)	1.50 (1.40–1.61)
Acute coronary syndrome	1.27 (1.09–1.48)	0.72 (0.63–0.81)	0.83 (0.70–0.97)

Odds ratios for AKI and AKR, based on changing sCr over 72h following admission. Mortality refers to death by day 30. The covariates that were used in the regression model are listed in the table. AKI: acute kidney injury; AKR: acute kidney functional recovery; RAS: renin-angiotensin system; SBP: systolic blood pressure.

## Results

Out of 81,351 patients hospitalized in the departments of Medicine, 53,289 patients were included in the study, having sCr values on admission- and repeated measurements within 72h from admission. Demographic and clinical characteristics of the entire cohort are outlined in Table 1. Out of these patients, 34,615 (65.5%) were managed by anti-hypertensive medications and/or loop diuretics. Noteworthy, as anticipated, this group of patients were older than the others, and had more co-morbidities, including CKD, hypertension, diabetes, heart failure, atrial fibrillation and acute coronary events. These patients also had higher blood pressure and lower heart rate, and their initial sCr and BUN were significantly higher. By contrast, acute infection was proportionally more common among patients not receiving anti-hypertensive medications or loop diuretics. As to medical management, RAS blocking agents were given to 36.7% of patients, over two thirds of them treated with ACEi, and the rest given ARBs. The in-hospital use of ARNI or renin inhibitors has been negligible. Loop diuretics were prescribed to 24% of patients, 43.4% were given beta blocking agents and 26% were treated with calcium channel blockers (CCB). As to clinical outcomes, the group managed by anti-hypertensive medications and diuretics had a significantly higher incidence of AKI, death and the need of dialysis, and reduced occurrence of AKR (*p* < 0.01) (Table 1).

Multivariate logistic regression models, exploring the association between anti-hypertensive medications and loop diuretics and the likelihood of developing AKI, AKR and mortality, are presented in Table 2. The administration of RAS blockers among inpatients was, unexpectedly, associated with a significant reduction in the chance of developing AKI (adjusted odds ratio 0.76, CI 0.70–0.83) and AKR (0.55, 0.52–0.58) and with a distinct decline in mortality (0.44, 0.41–0.48). By contrast, the use of loop diuretics was associated with an increased risk of AKI (adjusted odds ratio 3.75, 3.42–4.12), a declining chance of AKR (0.71, 0.66–0.75) and a higher likelihood of death (1.71, 1.58–185). Also illustrated in Table 2 are the odds ratios of the primary and secondary outcomes associated with patients’ characteristics. As anticipated [26,27], CKD, diabetes and acute infection were all associated with a substantially higher likelihood of developing AKI and AKR. Interestingly, increments in blood pressure were accompanied by a lower chance of AKI, AKR and mortality, while the use of beta-blockers predicted increased likelihood of AKI and decreased chance of AKR and mortality. Of note, age did not affect the impact of RAS blockade on the propensity to develop AKI or AKR: RAS inhibition reduced the likelihood for AKI (OR 0.76, 0.70–0.84) and AKR (OR 0.45, 0.41–0.48) among 31,427 patients aged >65 years, quite similar to the impact found for the entire cohort and among patients aged ≤65 years. Administering calcium channel blockers had no effect on AKI, AKR and mortality.

Descriptive statistics of a selected cohort of patients with advanced renal failure on admission (eGFR < 30 mL/min/1.73m^2^) are outlined in Table 3. Out of 6,270 patients (11.8% of the entire cohort), 4,955 (79%) were on antihypertensive medications and/or loop diuretics. The distribution pattern of comorbidities in this subgroup of patients with advanced CKD, equating drug-treated- and untreated patients, was comparable to that in the whole cohort of patients. Yet, fewer patients were given RAS inhibitors (24.8% as compared with 36.7% in the entire cohort), while a substantially larger proportion of patients were prescribed with loop diuretics and CCB. First sCr and BUN were modestly higher in the group of patients not given RAS blockers. As to the clinical outcomes, treatment with antihypertensive medications and loop diuretics was associated with a higher incidence of AKI and the need for dialysis, a lower occurrence of AKR, and, unexpectedly, a substantially lower mortality rate. Table 4 illustrates the outcome of multivariate logistic regression analyses, evaluating the association between anti-hypertensive medications and loop diuretics and the likelihood of developing AKI, AKR and mortality among those patients with advanced CKD. Overall the outcome patterns resemble those found in the entire cohort: Specifically, RAS inhibition was associated with roughly a 22% reduced probability of developing AKI or AKR and with a 45% lower chance of mortality. Hosmer-Lemeshow test for all logistic regression models demonstrated similar observed and expected frequencies across the groups (p-values > 0.05).

**Figure 1.**
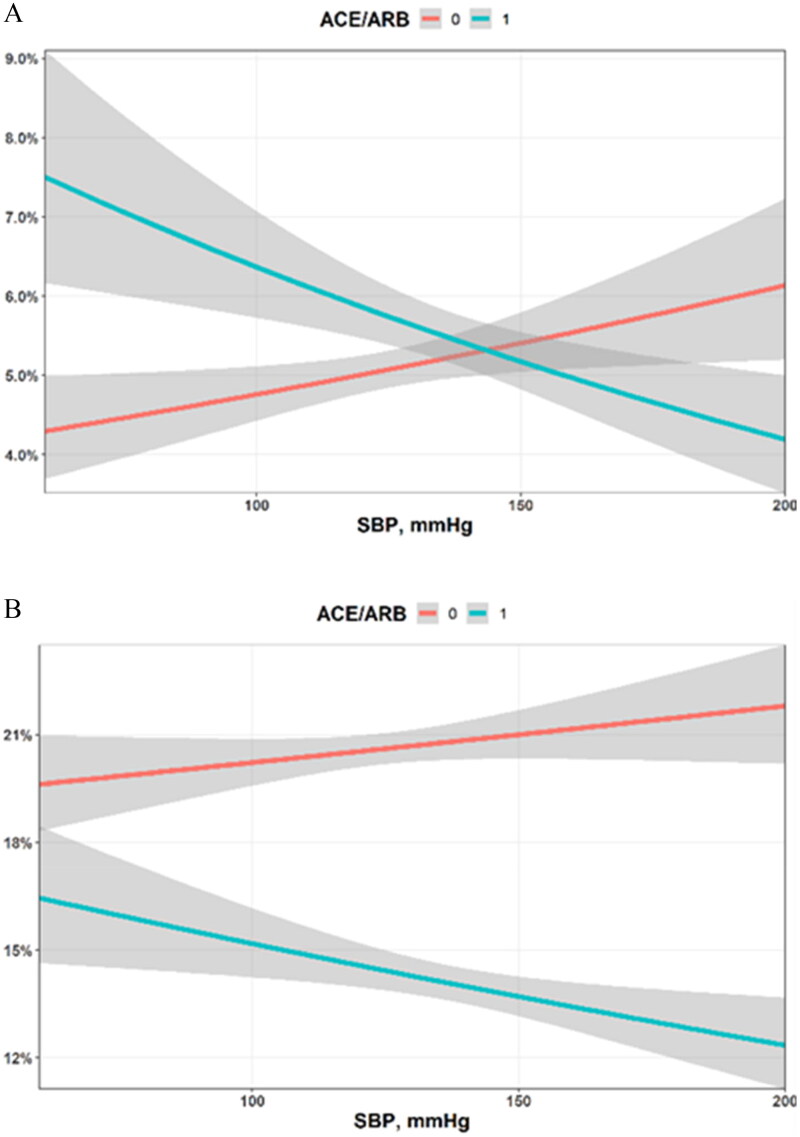
The impact of blood pressure on the likelihood to develop AKI (A) and AKR (B) in patients treated- or untreated with RAS inhibitors. Adjusted probabilities are shown for the first 72 h from admission, stratified by mean systolic blood pressure (SBP) along this period of time. RAS blockade enhances the probability of AKI at lowest SBP range, with a statistically insignificant 56% increased probability at SBP < 90 mmHg. By contrast, the impact of administered RAS blockers on AKR is evident across the entire range of SBP.

Figure 1 illustrate the impact of RAS inhibitors on the likelihood of developing AKI or AKR over 72 h from admission. Shown are the outcome in the entire cohort of 53,289 inpatients, adjusted for by the mean systolic blood pressure throughout the first three days of hospitalization. Evidently, RAS inhibition consistently reduced the adjusted probability of AKR across the entire spectrum of blood pressure measurements. By contrast, declining blood pressure measurements were associated with a trend to a higher risk of AKI in patients on RAS inhibitors. Among 376 patients with SBP < 90 mmHg, the odds ratio for AKI with the use of RAS inhibitors increased to 1.57, but with the small number of patients this figure did not reach statistical significance (CI 0.47–4.72). Patterns of regression lines for AKI and AKR across the scale of diastolic- and mean blood pressure measurements were comparable to those related to systolic blood pressure, depicted in Figure 1.

**Table 3.** Demographic and clinical characteristics of patients with advanced renal failure upon admission (eGFR < 30 mL/min/1.73m2), also stratified by the administration of anti-hypertensive medications and loop diuretics during hospitalization.

Variable	All patients (*n* = 6,270)	Anti-HTN/diuretic Tx (*n* = 4,955)	Control (*n* = 1,315)
Age, years (medians with inter quartile ranges)	79.4 [70.1, 86.4]	79.7 [70.9, 86.3]	78 [65.6, 86.5]
Male, n (%)	3,046 (48.5%)	2,382 (48%)	664 (50.5%)
Diagnoses, n (%)			
Diabetes mellitus type II	3,103 (49.4%)	2,665 (53%)	438 (33.3%)
Hypertension	4,581 (73%)	3,899 (78.9%)	682 (51.9%)
CHF	1,654 (26.3%)	1,560 (31.4%)	94 (7.1%)
Atrial fibrillation	1,318 (21%)	1,212 (24%)	106 (8.1%)
Multiple myeloma	74 (1.1%)	56 (1.1%)	18 (1.4%)
Acute infection	2,114 (33.7%)	1,475 (29.7%)	639 (48.6%)
Acute coronary syndrome	344 (5.4%)	323 (6.5%)	21 (1.6%)
Medications and interventions during hospitalization, n (%)			
RAS inhibition	1,556 (24.8%)	1,556 (31.4%)	0 (0%)
ACE inhibitors	998 (15.9%)	998 (20%)	
ARBs	610 (9.7%)	610 (12%)	
Renin inhibitor	2 (0%)	2 (0%)	
ARNI	7 (0.1%)	7 (0.1%)	
Beta blockers	3,381 (53.9%)	3,381 (68%)	0 (0%)
Loop diuretics	2,897 (46.2%)	2,897 (58.4%)	0 (0%)
CCB	2,544 (40.5%)	2,544 (51%)	0 (0%)
Contrast administration	390 (6.2%)	285 (5.8%)	105 (8%)
Packed red blood cells administration	597 (9.5%)	459 (9.3%)	138 (10.5%)
Vital signs (medians with inter quartile ranges)			
Systolic blood pressure, mmHg	130 [113, 150]	134 [116, 153]	120 [104, 135]
Diastolic blood pressure, mmHg	66 [60, 74]	67 [60, 74]	64 [57, 71]
Mean blood pressure, mmHg	87.8 [77, 99]	89.3 [78, 100]	82.6 [72, 92]
Oxygen saturation, %	96 [94, 98]	96 [94, 98]	97 [95, 98]
Heart rate, bpm	78 [69, 88]	76 [67, 86]	80 [76, 92]
Temperature, °C	37.3 [37, 37.9]	37.2 [37, 37.8]	37.5 [37.1, 38.2]
Laboratory results (medians with inter quartile ranges)			
Admission creatinine, mg/dL	2.6 [2.1, 3.5]	2.5 [2.1, 3.4]	2.8 [2.2, 4]
BUN, mg/dL	52.3 [38.5, 73.5]	51.3 [38.2, 71]	57 [39, 82.5]
Potassium, mmol/L	4.2 [3.8, 4.7]	4.3 [3.9, 4.7]	4.2 [3.7, 4.6]
Sodium, mEq/L	137 [134, 140]	137 [134, 140]	137 [134, 143]
Total protein, g/dL	6.2 [5.6, 6.7]	6.3 [5.7, 6.8]	5.8 [5.3, 6.4]
Albumin, g/dL	2.9 [2.5, 3.4]	3 [2.6, 3.4]	2.7 [2.2, 3.1]
CRP, mg/dL	13.3 [4.2, 30.9]	12.1 [3.7, 30.6]	16.8 [6.9, 31.8]
Blood pH	7.3 [7.3, 7.4]	7.3 [7.3, 7.4]	7.3 [7.2, 7.4]
Lactate, mmol/L	2.2 [1.5, 3.2]	2 [1.5, 3]	2.6 [1.8, 4.1]
Hemoglobin, mg/dL	10.3 [9, 11.8]	10.3 [9, 11.8]	10.3 [8.8, 11.9]
White blood cells, k/mcL	9.5 [7, 13.1]	9.2 [6.9, 12.3]	11.1 [7.7, 15.8]
Platelets, k/mcL	200 [150, 263]	201 [153, 262]	197 [140, 269]
Outcome, n (%)			
AKI grade 1	911 (14.5)	752 (15.2)	159 (12.1)
AKI grades >1	124 (2)	83 (1.7)	41 (2.1)
AKR grade 1	3513 (56)	2538 (51.2)	975 (74.1)
AKR grades >1	482 (7.7)	264 (5.3)	218 (16.6)
Dialysis within 30 days	121 (1.9)	108 (2.2)	13 (1)
Mortality within 30 days	1299 (20.7)	891 (18)	408 (31)

Blood pressure values represent medians of repeated measurements over 72h from admission. All other vital signs and laboratory results are those on admission. AKI: acute kidney injury; AKR: acute kidney functional recovery; RAS: renin-angiotensin system; CKD: chronic kidney disease; CHF: congestive heart failure; ACS: acute coronary syndrome; ACE: angiotensin converting enzyme; ARB: angiotensin receptor blocker; ARNI: angiotensin receptor-neprilysin inhibitor; CCB: calcium channel blockers; BUN: blood urea nitrogen; CRP: C-Reactive protein.

**Table 4.** Adjusted odds ratios of variables and interaction terms for the logistic regression models of acute kidney injury (AKI), acute kidney recovery (AKR) and mortality in 6270 patients with advanced CKD (eGFR < 30 mL/min/1.73m^2^).

GFR < 30			
Adjusted odds ratio (95% confidence interval)			
	AKI	AKR	Mortality
Beta blockers	0.94 (0.80–1.10)	0.85 (0.76–0.97)	0.64 (0.55–0.74)
RAS inhibitors	0.77 (0.64–0.92)	0.79 (0.70–0.89)	0.55 (0.46–0.65)
Loop diuretics	2.38 (2.02–2.82)	0.34 (0.30–0.38)	1.29 (1.11–1.51)
Contrast administration	1.53 (1.16–1.99)	1.05 (0.84–1.33)	1.15 (0.87–1.50)
Male gender	1.14 (0.97–1.30)	1.02 (0.92–1.14)	0.97 (0.85–1.11)
Age (per increase of 10 years)	0.93 (0.88–0.99)	0.95 (0.91–0.99)	1.04 (1.04–1.05)
Diabetes mellitus	1.09 (0.93–1.26)	1.02 (0.91–1.14)	1.04 (0.90–1.20)
Atrial fibrillation	0.98 (0.81–1.18)	0.91 (0.79–1.05)	0.97 (0.82–1.16)
CHF	0.96 (0.80–1.15)	0.87 (0.76–1.00)	1.02 (0.86–1.21)
Minimum SBP (per increase of 10 mmHg)	0.99 (0.98–0.99)	0.99 (0.98–0.99)	0.96 (0.96–0.97)
Acute infection	1.26 (1.08–1.47)	1.15 (1.02–1.30)	1.33 (1.16–1.53)
Acute coronary syndrome	1.63 (1.23–2.12)	0.66 (0.52–0.83)	0.95 (0.70–1.26)

Odds ratios for AKI and AKR, based on changing sCr over 72h following admission. Mortality refers to death by day 30. The covariates that were used in the regression model are listed in the table. AKI: acute kidney injury; AKR: acute kidney functional recovery; RAS: renin-angiotensin system.

## Discussion

We have recently explored the nature of early changes in sCr among inpatients following hospital admission, analyzing large datasets with propensity score matching and multivariate regression analyses [22,23,28]. Collectively, our findings revealed that AKI and AKR, defined by changing sCr, are closely co-associated along the scale of kidney function on admission, with progressively increasing likelihood to develop AKI or AKR as baseline kidney function on admission declines [24,26]. These findings favor the possibility that rising odds of developing AKI and AKR reflect diminishing renal functional reserve: as long as renal nephron mass is intact, acute functional dropout and subsequent recruitment of a fraction of the injured nephrons may go unnoticed, thanks to compensatory hyperfiltration of remnant nephrons (“subclinical AKI and AKR”). However, in CKD, as functional renal mass declines, renal functional reserve is lost, since remnant nephrons are already maximally hyperfiltrating, and cannot compensate for an additional transient or permanent nephron loss. This would lead to overt changes in sCr (“clinical AKI/AKR”) [27]. The rapid activation and de-activation of RFR is governed by the regulation of intraglomerular hemodynamics and filtration process, where the RAS plays a central role.

The interruption of the RAS axis interferes with such adaptive mechanisms by *a priori* efferent arteriolar vasodilation. This indeed reduces AKR capacity. On the other hand, RAS blockade is anticipated to exert renal vasodilation and to blunt Ang II-induced renal cortical hypoxia [29,30]. Furthermore, the attenuation of adaptive hyperfiltration is expected to decrease downstream oxygen consumption for tubular transport and attenuate medullary hypoxic stress under acute settings, preventing AKI. ARBs particularly may be renoprotective as medullary blood flow is maintained *via* angiotensin AT2 receptors, abundant in the outer medulla [31], and by Ang II-derived Ang 1-7, activating MasR, both exerting vasodilation [32]. Thus, RAS blockade is conceivably expected to prevent hypoxic AKI. Furthermore, it is not supposed to lead to a substantial decline in GFR, unless it heavily depends on renal perfusion pressure as happens with volume depletion and utmost efferent arteriolar vasoconstriction or in renal artery stenosis with remnant nephrons maximally activating their functional reserve.

Our current findings, shown in Table 2, indicate that among inpatients in medical wards, reduced SBP, RAS inhibition and loop diuretics substantially affect the odds of developing AKI or AKR. Outstandingly, RAS blockers were found to be associated with reduced incidence of AKI and AKR and with improved survival. By contrast, administration of loop diuretics was found to be associated with an increased incidence of AKI, reduced likelihood of AKR and increased mortality. Furthermore, we found that markedly reduced BP increases the odds of developing AKI in patients on RAS inhibitors. These effects on AKI and AKR likely occur through disruption of trans-glomerular pressure and intervening with auto-regulatory components operating RFR. Most importantly, the reduced odds of developing AKI among patients of RAS blockade is also evident in patients with advanced renal failure, as shown in Table 4, consolidating the conclusion that as long as systemic blood pressure is maintained, RAS inhibition is safe and even renoprotective in hospitalized patients with acute medical conditions, irrespective of their kidney function. Prevention of AKI by RAS inhibition in patients with advanced CKD is conceptually in line with evidenced safe long-term use of RAS inhibitors in such patients, even suggesting a trend for retarding the initiation of renal replacement therapy [33]. In that respect, the reduced likelihood of AKR in patients on RAS inhibitors should not be regarded as worrisome, since it may reflect a lower rate of AKI on admission with subsequent recovery.

Our findings reveal an increased likelihood of AKI and AKR in older patients and among those with CKD, diabetes, and lower blood pressure (Table 2), fitting well with our concept linking AKI and AKR with declining RFR. As an example, age-related reduced renal functional mass and RFR unmask abrupt up and down changes in GFR, usually compensated for by non-injured nephrons [24,27]. Acute infection is also linked to an increased risk of AKI and AKR, while the use of diuretics is associated with increased risk of AKI and hampered AKR capacity, likely highlighting the importance of euvolemia. Furosemide attenuates tubuloglomerular feedback and blocks NaK2Cl co-transporter in medullary thick limbs. Though it exerts profound renal vasoconstriction, medullary hypoxia is attenuated by the inhibition of oxygen consumption for tubular reabsorption. Indeed, furosemide attenuates hypoxic outer medullary injury ex-vivo [34] and in vivo [35]. Thus, the profound increased odds of AKI and reduced likelihood of AKR, associated with furosemide, shown in Table 2, underscores the likely predominant component of pre-renal failure, but without biomarker data we can only speculate on the existence of concomitant tubular injury. The use of beta-blocking agents was also associated with increased risk of AKI and reduced odds of AKR (Table 2), interestingly despite improved renal parenchymal oxygenation, demonstrated in hypertensive patients with suspected renal artery stenosis [36]. The nature of this association, therefore, remains speculative.

The most unexpected outcome was the marked 56% decline in the likelihood of 30-day mortality in patients on RAS blocking agents during hospitalization (Table 2). This association was also evident in patients with advanced CKD, where the odds of mortality declined by 45% (Table 4). This association was noted among 31,427 patients aged >65 years (OR 0.45, 0.41–0.48) and among the 21,862 patients aged ≤65 years (OR 0.41, 0.32–0.51). Our data base does not specify cause of death and whether RAS inhibition was extended beyond discharge. Further studies are therefore required to consolidate this observation and to assess possible mechanisms. Yet, our findings are in line with previous propensity-matched studies [7,8,10], and with an observational large retrospective study, unrelated to acute illnesses, showing an association between withholding RAS inhibitors in patients with advanced CKD and an enhanced likelihood of major cardiovascular events and mortality [37].

The very large cohort of heterogenous population included in this study with the wide spectrum of diseases characterizing inpatients in departments of Medicine forms the strength of this study. The drawbacks of the present study include its retrospective non-controlled nature, the small fraction of patients with risk factors, particularly heart failure, in the group not on RAS inhibition, and the possibility of unrecognized CKD by patient’s report on admission. Even though we used multivariate regression analyses, this study may be especially subject to indication bias (also known as confounding by indication), as medical care providers likely consider the risk of AKI when prescribing RAS blockade to acutely ill and hemodynamically unstable patients. This limitation is nevertheless somewhat adjusted for by the correction for blood pressure, as illustrated in Figure 1. Our analysis also does not address changes in the dosing of RAS blockers and diuretics within the first 72 h following admission. Additionally, this is a single-center study that may not represent diverse populations. It also does not include patients in critical care units, though over 10% of included patients were managed in advanced treatment units for critically ill patients within Medical departments. By and large, in-hospital prescribing RAS inhibitors was a continuance of pre-admission treatment, but we have unreliable data regarding pre-hospital consumption of RAS inhibitors and we cannot comment on the possible impact of withholding these drugs upon admission. Our data base also cannot address the impact of SGLT-2 inhibitors since their use was almost mandatorily withheld under settings requiring hospitalization, with concern regarding the risk of AKI [38].

In summary, the principal operative conclusion of this study is that as long as blood pressure is maintained, RAS inhibition seems to be safe and even renoprotective in hospitalized patients with acute medical conditions, irrespective of their estimated kidney function on admission. It also has a substantial association with a better survival, with a 56% reduced mortality by day 30. Additional studies are needed to confirm and consolidate these conclusions, utilizing propensity score matching or target trial emulation, in order to exclude a possible confounding impact of biased selection of patients regarding the continuation or interruption of RAS inhibitors upon hospital admission. Further studies are also needed, extending the evaluation to critically ill patients and to other ethnic groups, and looking at the impact of RAS inhibitors specifically in subsets of diverse acute critical illnesses.
